# Polyphyllin B Suppresses Gastric Tumor Growth by Modulating Iron Metabolism and Inducing Ferroptosis

**DOI:** 10.7150/ijbs.80324

**Published:** 2023-01-31

**Authors:** Can Hu, Dan Zu, Jingli Xu, Hangdong Xu, Li Yuan, Jiahui Chen, Qin Wei, Yanqiang Zhang, Jing Han, Tao Lu, Jinyun Dong, Jiang-Jiang Qin, Zhiyuan Xu, Xiangdong Cheng

**Affiliations:** 1 Department of Gastric surgery, Zhejiang Cancer Hospital, Institutes of Basic Medicine and Cancer (IBMC), Chinese Academy of Sciences, Hangzhou 310022, China.; 2 Department of Integrated Chinese and Western medicine, Zhejiang Cancer Hospital, Institutes of Basic Medicine and Cancer (IBMC), Chinese Academy of Sciences, Hangzhou 310022, China.; 3 Department of Abdominal Oncology, Zhejiang Cancer Hospital, Institutes of Basic Medicine and Cancer (IBMC), Chinese Academy of Sciences, Hangzhou 310022, China.; 4 Biospecimen Repository, Cancer Hospital of the University of Chinese Academy of Sciences.; 5 Key Laboratory of Prevention, Diagnosis and Therapy of Upper Gastrointestinal Cancer of Zhejiang Province, Hangzhou 310022, China.; 6 Zhejiang Provincial Research Center for Upper Gastrointestinal Tract Cancer, Zhejiang Cancer Hospital, Hangzhou 310022, China.

**Keywords:** Gastric cancer, GPx4, ferroptosis, iron ion metabolism, natural product

## Abstract

Gastric cancer (GC) is one of the most common malignant tumors in the world. GPx4, as the core regulator of ferroptosis, has become a potential molecular target for developing anticancer agents. In the present study, we found that GPx4 was overexpressed and negatively correlated with poor prognosis in GC, while it was associated with the GC development. Molecular docking and structure-based virtual screening assays were used to screen potential GPx4 inhibitors, and we identified a novel GPx4 inhibitor, polyphyllin B (PB), which can induce ferroptosis by down-regulating GPx4 expression in GC cells. It has also been shown to inhibit cell proliferation, suppress invasion and migration, induce apoptosis, and block the cell cycle progression in GC cells *in vitro*. Then, immunofluorescence and western blotting assay confirmed that PB can regulate the expression of LC3B, TFR1, NOCA4 and FTH1 *in vitro*, which suggested that suggest that PB may increase the level of Fe^2+^ by transporting Fe^3+^ into the cell by TFR1 and promoting NCOA4-dependent iron autophagy. In addition, PB can also suppresses tumor growth in an orthotopic mouse model of GC via regulating the expression of GPx4, TFR1, NOCA4 and FTH1 *in vivo*. In summary, we confirmed that GPx4 may be a potential target for GC treatment, PB may be a novel and promising drug for the treatment of GC, which shows good antitumor efficacy without causing significant host toxicity via inducing ferroptosis in both gastric cancer cells and mouse models.

## Introduction

Gastric cancer (GC) is one of the most common malignant tumors in the world and a serious threat to human health. According to the latest report of the International Agency for Research on Cancer (IARC), there were approximately 1.03 million new patients with GC in the world in 2018 1. The 5-year survival rate of patients with early GC is more than 90%, while the 5-year survival rate of patients with advanced GC is less than 30% 2. In recent years, with the development of molecular targeted therapy and immunotherapy, the prognosis of non-small cell lung cancer, breast cancer and other malignant tumours has significantly improved. However, these new treatments did not result in obvious survival benefits for patients with GC.

Glutathione peroxidase 4 (GPx4), a selenocysteinase, is a member of the glutathione peroxidase family. It can reduce toxic lipid peroxide to nontoxic lipid alcohols in the presence of reduced glutathione (GSH), thus preventing ferroptosis 3-5. In recent years, an increasing number of studies have found that GPx4 plays an important role in a variety of common malignancies. High expression of GPx4 was detected in breast cancer 6, liver cancer 7, lung cancer 8 and glioma 9, suggesting that GPx4 may play a role as an oncogenic gene. In addition, GPx4 can also promote tumour proliferation, invasion and metastasis by maintaining stemness.

GPx4 is the core regulator of ferroptosis, and direct targeting of GPx4 to induce ferroptosis in cancer cells is a potential strategy for developing anticancer agents. RSL3, the first inhibitor found to directly inhibit GPx4, contains an electrophilic chloroacetamide that binds irreversibly to its active site, leading to GPx4 inactivation 10. Other GPx4 inhibitors have since been identified, including ML162, DPI compounds (DPI includes DPI7, 10, 12, 13, 17, 18, and 19), FIN56, and FINO2 11, 12. However, most of the existing GPx4 inhibitors have poor pharmacokinetic properties, which limits their clinical application. Its active components play an important role in promoting ferroptosis in tumor cells. Chen et al. 13 confirmed that β-elemene extracted from natural materials can induce ferroptosis, regulating epithelial-mesenchymal transition in colorectal cancer.

In this study, we explored the expression of GPx4 in GC and identified a small molecule GPx4 inhibitor, polyphyllin B (PB), and investigated its anti-GC activity and mechanism in GC models* in vitro* and* in vivo*. Our results suggest that PB may be a potential anti-GC drug.

## Methods

### Cell lines and chemicals

The GC cell lines NUGC-3, MKN-1, MKN-45, HGC-27, and NUGC-4 were obtained from Shanghai Bioleaf Biotech Co., Ltd. (Shanghai, China). Cells were cultured in RPMI 1640 medium (Kino Biological and Pharmaceutical Technology Co., Ltd., Hangzhou, China) containing 10% foetal bovine serum (FBS, Gibco, Grand Island, USA) and 1% penicillin/streptomycin (Kino Co., Ltd., Hangzhou, China) in a humidified atmosphere containing 5% CO2/95% air at 37 °C. The antibodies anti-GPx4 (Cat No. ab125066), anti-CDK4 (Cat No. ab108357), anti-CDK4 (Cat No. ab124821), anti-Cyclin D1 (Cat No. ab16663), anti-Ferritin Heavy chain (Cat No. ab287968) and anti-TFRC (Cat No. ab214039) were purchased from Abcam (Cambridge, UK). Anti-LC3B (Cat# 2775) antibody was purchased from Cell Signaling Technology (Beverly, MA, USA). Anti-NCOA4 (Cat. Sc-373739) was purchased from Santa Cruz Biotechnology (Texas, USA).

### H&E and immunohistochemistry

The normal gastric tissues, intestinal metaplasia tissues, dysplasia tissues, early GC tissues, and advanced GC tissues were collected from Zhejiang Cancer Hospital. And the H&E-stained slides from each FFPE block were evaluated by two pathologists. For IHC, the normal gastric tissues, intestinal metaplasia tissues, dysplasia tissues, early GC tissues, and advanced GC tissues were stained with antibodies against GPx4 (Abcam, ab125066,1/100), followed by Goat Anti-Rabbit IgG H&L (HRP) (Abcam, ab6721,1/200). The results were evaluated by two pathologists independently via microscope (Olympus BX43).

### Molecular docking

Molecular docking and virtual screening were performed with AutoDock Vina software as described previously 14-16. The crystal structure of GPx4 (PDB code: 6HKQ) was retrieved from the Research Collaboratory for Structural Bioinformatics Protein Data Bank (RCSB PDB, http://www.rcsb.org/pdb/). The PB standard structure (PubChem CID: 21603986) was retrieved from the PubChem Compound Database (https://pubchem.ncbi.nlm.nih.gov/). The grid parameters of GPx4 were identified as centre_x, -24.581; centre_y, 9.304; and centre_z, 2.804, and the dimensions were size_x, 15.879; size_y, 15.879; and size_z, 15.879. The default parameters were used unless otherwise mentioned. The best-fit complex was visually analysed with Chimaera 1.14 and PyMOL 2.4.0 software.

### Cell viability assay

The cell viability assay was performed as previously described 17. Briefly, 2000-3000 cells/well were seeded in a 96-well plate overnight and then exposed to PB (1.0, 2.0, 3.0, 4.0, 5.0, 6.0, 8.0, 10.0 µM) or DMOS for 24 h. At each time point, 10 µl CCK-8 solution (Dojindo) was added to each well and incubated for another 1 h at 37 °C. The absorbance at 450 nm was determined using a microplate reader. The cell viability and IC_50_ values were calculated.

### Colony formation assay

Approximately 800 MKN-1 and NUGC-3 GC cells were seeded in a 6-well plate overnight and treated with PB (0.5, 1.0 or 2.0 µM) or DMSO for 24 h. Then, the cells were cultured with RPMI-1640 medium for 15 days. Finally, the cells were fixed and stained with crystal violet (Solarbio, China).

### Transwell experiments

Approximately 3×10^4^ MKN-1 and NUGC-3 GC cells were suspended in 200 µL serum-free medium and inoculated in the upper compartment of a Transwell chamber (Corning, USA) in the presence of PB (1.0 or 2.0 µM) or DMSO. Furthermore, 500 µL of complete medium containing 20% FBS was added to the lower chamber. After 48 h, the cells on the upper surface of the cell membrane were removed with sterile cotton swabs and washed with PBS. The rest of the plates were stained with crystal violet (Solarbio, China) and analysed under a microscope (Axio observer A1, Zeiss, Germany).

### Wound healing assay

Approximately 3 × 10^5^ MKN-1 and NUGC-3 GC cells were seeded in a 6-well plate. After the cell filled the entire area, the culture inserts were removed. The cells were treated with PB (1.0 or 2.0 µM) or DMSO for 48 h. The cells were then rinsed twice with PBS to remove floating cells. Photos were taken under an optical microscope (ix71, Olympus, Japan) at 0 h, 24 h and 48 h after wound induction.

### Western blotting

Cells were lysed, and total protein was extracted. Western blotting was performed as previously described 17. The protein was separated by sodium dodecyl sulfate-polyacrylamide gel electrophoresis (SDS‐PAGE) and then transferred to polyvinylidene fluoride (PVDF) membranes. The membranes were blocked with 5% skim milk and then incubated with primary antibodies overnight at 4 °C. The membranes were then incubated with appropriate secondary antibodies (Cell Signaling Technology) for 2 hours after being washed with Tris‐buffered saline with Tween (TBST). Finally, the bands were visualized by enhanced chemiluminescence (ECL; Thermo-Pierce, Rockford, IL, USA). Data were calculated and normalized to β-actin.

### Immunofluorescence

Approximately 5 × 10^4^ MKN-1 and NUGC-3 GC cells were seeded in 15 cm dishes overnight and treated with PB for 24 h. The culture medium was removed, and the cells were washed with precooled PBS and fixed with 4% paraformaldehyde for 20 min. The membrane was permeabilized with 0.5% PBST for 30 min after cleaning, followed by blocking with immunofluorescence blocking solution for 1 h. NCOA4 and FTH1 antibodies were added to the 24-well plate for incubation at 4 °C overnight. Alexa Fluor 488-labelled goat anti-rabbit IgG antibody was added to the 24-well plate for incubation for 1 hour at room temperature. Then, LC3B antibody was added to the 24-well plate for incubation at 4 °C overnight. Alexa Fluor 594-labelled goat anti-mouse IgG antibody was added to the 24-well plate for incubation for 1 hour at room temperature. The climbing cells were carefully removed from the 24-well plate in the dark. A laser confocal microscope was used to observe the cells and take micrography.

### Cell apoptosis and cell cycle assays

Approximately 3 × 10^5^ MKN-1 and NUGC-3 GC cells were seeded in a 6-well plate overnight and treated with PB for 24 h. Apoptosis assays were performed by FACS using an Annexin V-FITC/PI Staining Kit (LiankeBio, Hangzhou) following the manufacturer's instructions. The cell cycle was analysed with a Cell Cycle and Apoptosis Analysis Kit (Biosharp, Beijing) following the manufacturer's instructions.

### Cellular thermal shift assays

Approximately 2 × 10^8^ MKN-1 and NUGC-3 GC cells were seeded in 15 cm dishes overnight and treated with PB for 1 h. GC cells were collected, centrifuged, resuspended in precooled PBS with 1% protease inhibitor, and transferred to PCR tubes. Then, the PCR tubes were heated at the specified temperature for 3 min in a PCR amplifier, immediately removed and incubated at room temperature for 3 min, after which the samples were cooled in liquid nitrogen for 3 min. Finally, the protein was detected and quantified by western blotting analysis.

### Lipid peroxidation assay

MKN-1 and NUGC-3 GC cells were seeded in 6-well plates and treated with different concentrations of PB (0.5, 1.0 or 2.0 µM) or DMSO for different times (2, 4 or 8 h). Then, GC cells were collected and resuspended in 500 µL PBS with 2 µmol/L C11-BODIPY^581/591^ (Thermo Fisher Scientific, USA) for 30 min at 37 °C. Then, GC cells were measured by flow cytometry (Agilent, USA). Finally, the quantification of the data was analysed by FlowJo (Mac OSX 10.4 version).

### GPx activity assay

Approximately 2000-3000 cells/well were seeded in a 96-well plate overnight and then exposed to PB (0.5, 1.0 or 2.0 µM) or DMOS for 24 h. According to the manufacturer's instructions, GPx activity was detected using the Glutathione Peroxidase Assay Kit (Abcam, Cat No. ab102530).

### Fe^2+^ intensity assay

Approximately 2000-3000 cells/well were seeded in a 96-well plate overnight and then exposed to PB (0.5, 1.0 or 2.0 µM) or DMOS for 24 h. According to the manufacturer's instructions, the Fe^2+^ level was detected using the Iron Assay Ki (Abcam, Cat No. ab83366).

### RNA sequencing and bioinformatic analysis

MKN-1 GC cells were treated with 1.0 µM PB or the same volume of dissolvent for 24 h. Then, cell samples were collected and added to TRIzol reagent (Invitrogen, CA, USA) and then handed over to GeneChem Biotechnology Company (Shanghai, China) for subsequent mRNA library construction and sequencing. Bioinformatic analysis was performed as previously described. Hisat2 and Ballgown were used to compare the filtered CleanData with the human genome and to perform initial assembly and FPKM quantification of genes or transcripts. The differentially expressed mRNAs were selected with |log2FC|> 0.58 and *FDR* < 0.05 by the R package edgeR. The differentially expressed genes were selected to perform GO enrichment and KEGG enrichment analyses using DAVID. The genes associated with ferroptosis, as defined by the ferroptosis gene set in the GSEA database (M39768), were selected to generate a heatmap. Three replicates were performed for each group.

### Quantitative proteome analysis

MKN-1 GC cells were treated with 1.0 µM PB or the same volume of dissolvent for 24 h. Then, cell samples were collected and sent to GeneChem Biotechnology Company (Shanghai, China) for quantitative proteome library construction and sequencing. Then, protein extraction, protein quantification, SDS-PAGE, protein enzymatic hydrolysis and mass spectrometry detection were performed. The MS data were analysed using MaxQuant software (version 1.6.17.0). MS data were searched against the database. The cut-off of the global false discovery rate (FDR) for peptide and protein identification was set to 0.01. Protein abundance was calculated on the basis of the normalized spectral protein intensity (LFQ intensity). Proteins with a fold change>1.5 and p value (Student's t test) <0.05 were considered to be differentially expressed proteins. The differentially expressed proteins were selected to perform GO enrichment and KEGG enrichment. The genes associated with ferroptosis according to the ferroptosis gene set in the GSEA database (M39768) were selected to generate a heatmap. Three replicates were performed for each group.

### Orthotopic GC tumours in nude mice

BALB/c nude mice (male, 4 weeks old) were obtained from the experimental animal ministry of Zhejiang Chinese Medical University. All animal studies (including the mouse euthanasia procedure) were performed in compliance with the regulations and guidelines of Zhejiang Chinese Medical University institutional animal care and conducted according to the AAALAC and IACUC guidelines.

The nude mice were raised in our laboratory for a week before the experiment. Then, 5×10^6^ MKN-1 cells were subcutaneously injected to establish the subcutaneous xenograft tumour model in nude mice. When the maximum diameter of the xenograft tumours grew steadily to 1 cm, they were dissected completely and cut into 1 mm^3^ tissue fragments. Then, the tissue fragment was inserted into the surface of the serosa on the greater curvature of the stomach. Different doses of PB (2.5 mg/kg or 5.0 mg/kg) were given by intraperitoneal injection once a day for 3 weeks. The control group was given the same volume of vehicle. The positive control group was given 5-Fu at the dose of 10 mg/kg. The body weight and tumour size of nude mice were recorded. Mice were administered fluorescein substrate (150 mg/kg) intraperitoneally for *in vivo* imaging twice a week on a Xenogen IVIS 200 imaging system (Caliper Life Sciences, USA). The tumour inhibition rate was analysed using LT Living Image 4.3 Software.

## Results

### GPx4 overexpression was associated with poor prognosis in GC

**Figure 1:**
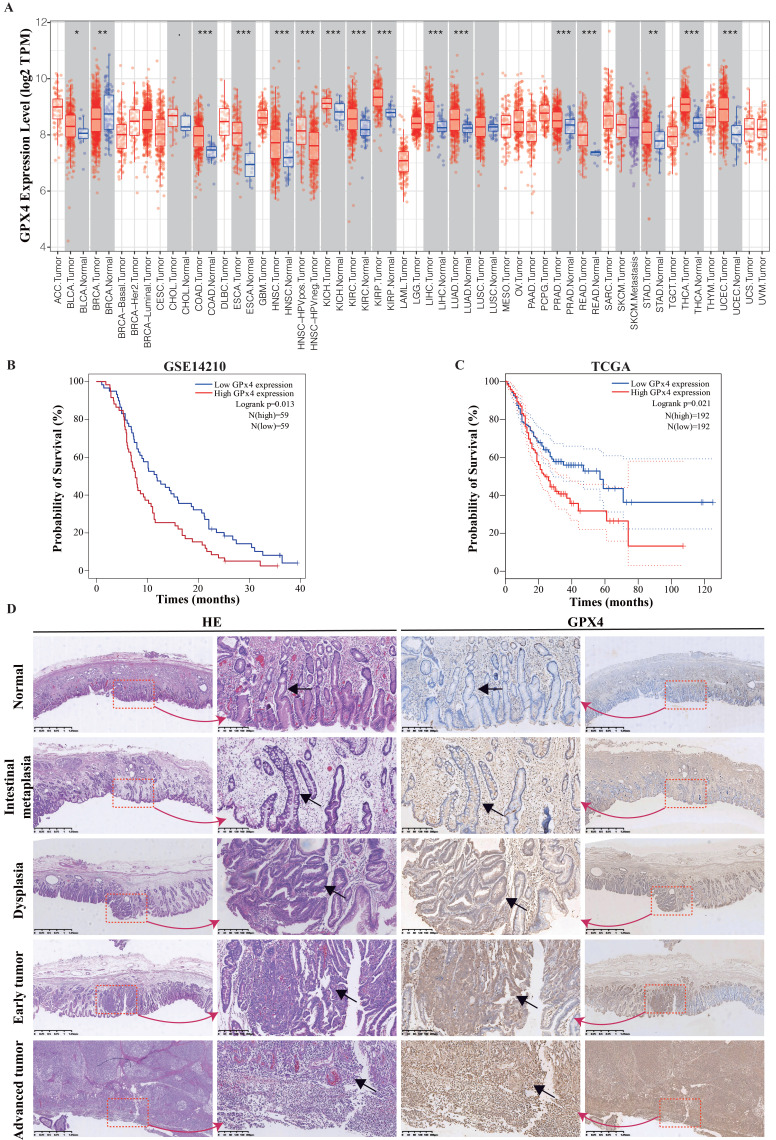
** GPx4 is overexpressed in GC and correlated with the evolution and poor prognosis. A:** The mRNA expression of GPx4 in Pan-cancer in TIMER database. **B and C:** Survival analysis for the relationship between GPx4 expression and overall survival of GC patients in the TCGA dataset and GES14210 dataset. **D:** Immunohistochemical analysis for the relationship between GPx4 expression and evolution of GC.

Using the TIMER database (TIMER1.0, https://cistrome.shinyapps.io/timer/), the mRNA expression levels of GPx4 between tumour and adjacent normal tissues in a pan-cancer dataset were confirmed (Figure 1A), and GPx4 was overexpressed in stomach adenocarcinoma compared with adjacent normal tissues (P<0.01). Survival analysis of GPx4 was performed in the GSE 14210 and TCGA cohorts (Figure 1B-1C). Patients with high GPx4 expression had a poorer prognosis than those with low GPx4 expression. Moreover, we also explored the level of GPx4 expression in the evolution of GC. The immunohistochemical results showed that the expression of GPx4 was gradually increased in normal tissues, intestinal metaplasia, dysplasia, early GC, and advanced GC (Figure 1D).

### Polyphyllin B was identified as a new small molecule GPx4 inhibitor

**Figure 2:**
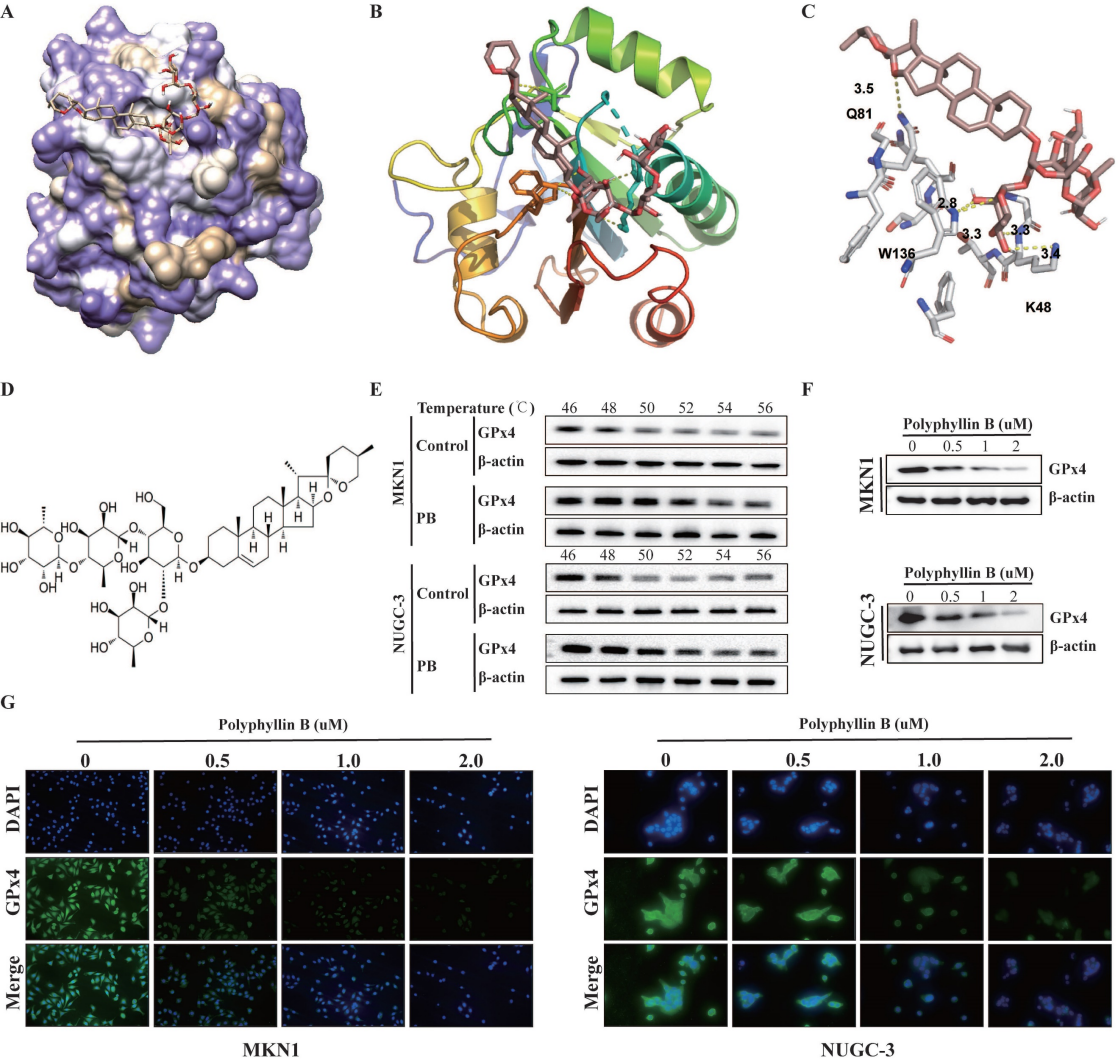
** PB directly targets GPx4. A, B and C:** Docking analysis for predicting the binding mode of PB to GPx4. **D:** Chemical structure of PB.** E:** Cellular thermal shift assays and western blot were used for evaluating the binding of PB and GPx4 in MKN-1 and NUGC-3 GC cells. **F-G:** Western blot and immunofluorescence were used for examined the GPx4 expression after PB treatment in MKN-1 and NUGC-3 GC cells.

To identify potential new small-molecule GPx4 inhibitors, we performed a docking-based virtual screening of an in-house library of natural products using the crystal structure of GPx4, and a number of new chemotypes of potential GPx4 inhibitors were identified. Among them, polyphyllin B (PB) was characterized as the most promising based on a preliminary GPx4 inhibitory evaluation. To rationalize the observed efficiency of GPx4 inhibition by the compound PB, we analysed the plausible binding modes. The molecular docking results showed that PB binds effectively and directly to the active pocket of GPx4 through the formation of five hydrogen bonds (see yellow dashed lines, Figure 2A-C) with the W136, K48 and Q81 residues at distances of 2.8 Å to 3.5 Å. Additionally, it can be speculated that the glycol unit of the side chain may be an important pharmacophore for GPx4 inhibition because this part was well embedded into the active site and interfered with the catalytic function of selenocysteine at position 46. Furthermore, the results of cellular thermal shift assays showed that the GPx4 protein disappeared with increasing temperature in the control group. However, a strong GPx4 band was detected as the temperature increased to 56 °C in the PB group (Figure 2E-F). Moreover, we found that PB downregulated the expression of GPx4 in a concentration-dependent manner (Figure 2G-H). This finding may suggest that PB effectively targeted GPx4 in both MKN-1 and NUGC-3 GC cells.

### Polyphyllin B exerts anticancer activities in GC cells *in vitro*

**Figure 3:**
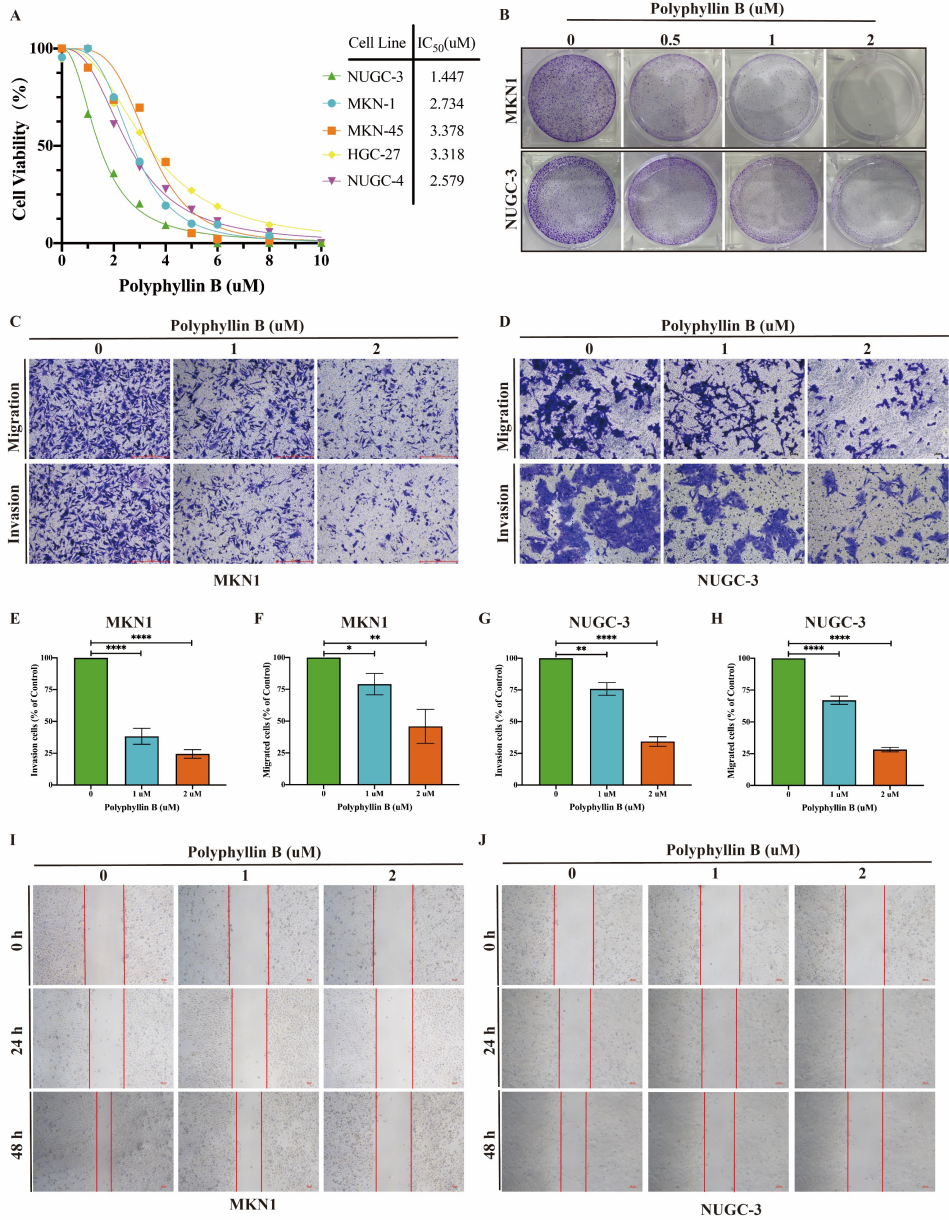
** PB inhibits GC cell proliferation, prevents migration and invasion in GC cells *in vitro*. A:** CCK8 assays was used for detecting the effect of PB on viability of human GC cell lines.** B:** The colony-formation assays were performed in MKN-1 and NUGC-3 GC cells. **C-F:** Transwell migration and invasion assays were performed after PB treatment for 48h in MKN-1 and NUGC-3 GC cells, followed by quantitative analysis. **G-H:** Quantitative analysis of wound healing assays after PB treatment for 24h and 48h in MKN-1 and NUGC-3 GC cells. *P < 0.05, **P < 0.01, *** P < 0.001, **** P < 0.0001.

**Figure 4:**
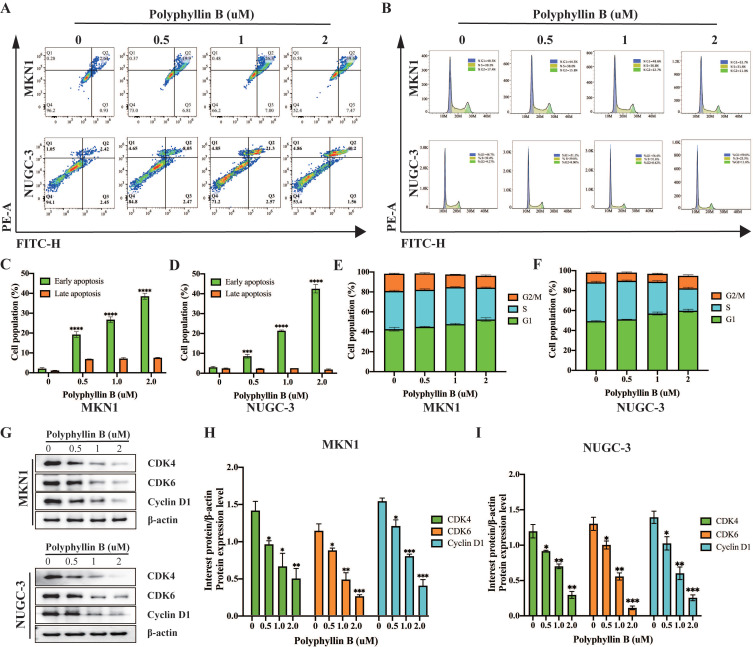
** PB promotes GC cell apoptosis and blocks cell cycle. A, C and D:** Representative results of Annexin V-FITC/PI staining and quantitative analysis after the PB treatment for 24 h in MKN-1 and NUGC-3 GC cells. **B, E and F:** Representative results of cell cycle and quantitative analysis after the PB treatment for 24 h in MKN-1 and NUGC-3 GC cells. G-I: The expression of CDK4, CDK6, Cyclin D1 were analyzed via western blot after the PB treatment for 24 h in MKN-1 and NUGC-3 GC cells, followed by quantitative analysis. * P < 0.05, ** *P* < 0.01, *** P < 0.001, **** P < 0.0001.

To examine the inhibitory effect of PB on the viability of GC cells, we treated five GC cell lines, namely, NUGC-3, MKN-1, MKN-45, HGC-27 and NUGC-4, with different concentrations of PB. We found that PB significantly inhibited the growth of GC lines (IC_50_ = 1.447-3.318 μM) in a concentration-dependent manner (Figure 3A). Colony formation assays showed that PB significantly reduced the efficiency of colony formation in MKN-1 and NUGC-3 GC cell lines (Figure 3B). Transwell experiments and wound healing assays showed that PB significantly prevented the migration and invasion of MKN-1 and NUGC-3 GC cell lines (Figure 3C-J). Moreover, we analysed the effect of PB on cell apoptosis and the cell cycle. The results showed that PB can induce cell apoptosis and block the cell cycle of MKN-1 and NUGC-3 GC cells in a concentration-dependent manner (Figure 4A-F). PB reduced the expression of CDK4, CDK6 and Cyclin D1 in MKN-1 and NUGC-3 GC cells (Figure 4G-I).

### Polyphyllin B induces ferroptosis in GC cells *in vitro*

**Figure 5:**
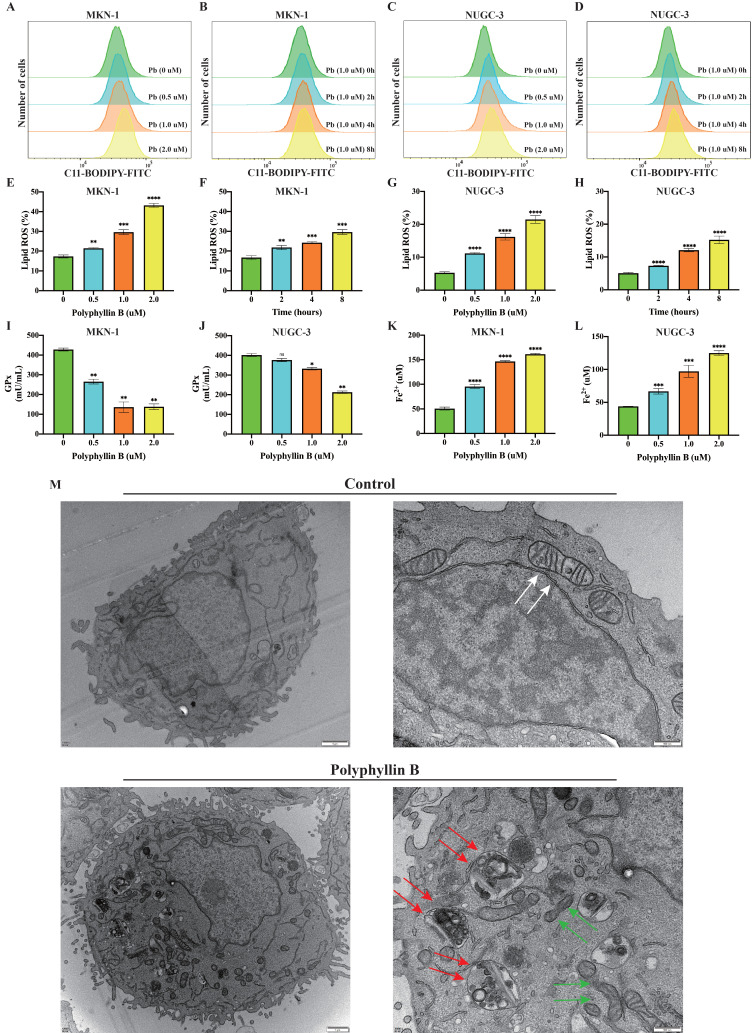
** PB induced ferroptosis in GC cells. A-H:** Lipid peroxidation assay were performed after different concentration or time PB treatment in MKN-1 and NUGC-3 GC cells, followed by quantitative analysis. **I-J:** GPxs assays were performed after PB treatment for 24h. K-L: Fe^2+^ assays were performed after PB treatment for 24h. **M:** Electron microscope was used to observe the cell structure after PB treatment for 24h. The white arrow showed mitochondrial morphology of untreated cells, green arrow showed mitochondrial morphology of PB treated cells, red arrow showed PB-induced autophagic vesicle. * P < 0.05, **P < 0.01, *** P < 0.001, **** P < 0.0001.

To examine whether PB can induce ferroptosis in GC cells. The level of lipid peroxidation, the activity of GPxs and the level of Fe^2+^ were analysed after PB treatment of the MKN-1 and NUGC-3 GC cell lines. The results showed that the level of lipid peroxidation was increased after PB treatment in a concentration- and time-dependent manner (Figure 5A-H). The GPx activity declined after PB treatment, while the level of Fe^2+^ increased (Figure 5I-K). Mitochondrial atrophy and mitochondrial ridges were decreased, while some autophagic vacuoles were observed under the electron microscope after PB treatment (Figure 5M). These results suggest that PB can induce ferroptosis in GC cells *in vitro*.

### Transcriptome analysis revealed significant changes at the mRNA level in genes related to ferroptosis after PB treatment

**Figure 6:**
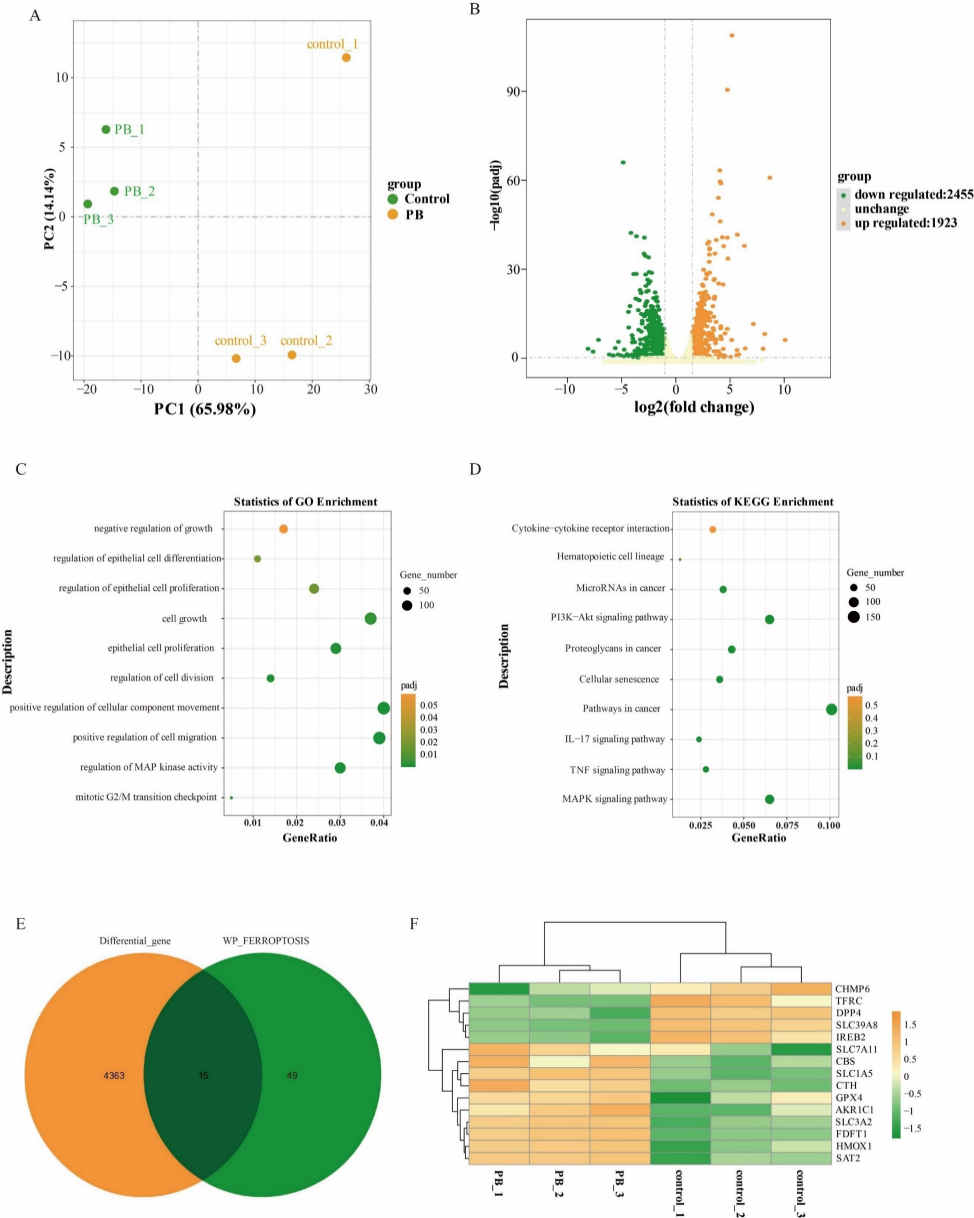
** Transcriptome sequencing analysis after PB treatment in MKN-1 GC cells. A:** PCA of transcriptomes with or without PB treatment represented in a two-dimensional space.** B:** Volcano plot showed the differentially expressed genes between PB-treated and untreated MKN-1 GC cells. **C-D:** GO analysis and KEGG analysis for the significantly differentially genes after PB treatment in MKN-1 GC cells. **E-F:** Venn diagram and heat map to show the differentially expressed genes associated with ferroptosis.

To investigate the molecular mechanism of how PB inhibits GC cells, RNA sequencing was performed on MKN-1 GC cells after PB treatment using DMSO treatment. After preliminary screening, a total of 4378 genes were identified as differentially expressed genes. Compared with the control group, 1923 genes were upregulated and 2455 genes were downregulated in the PB group (Figure 6B). To understand the effect of differential genes on GC cells, GO and KEGG analyses were performed. GO analysis showed that the differentially expressed genes after PB treatment were mainly involved in cell migration, cell division, epithelial cell differentiation, epithelial cell differentiation and cell growth, while KEGG analysis showed that PB can regulate the MAPK signalling pathway, TNF signalling pathway, IL-17 signalling pathway, and PI3K-Akt signalling pathway as well as cytokine‒cytokine receptor interaction (Figure 6C-D). This further confirmed the inhibitory effect of PB on MKN-1 GC cells. To fully understand the role of PB in regulating ferroptosis, the differentially expressed genes associated with ferroptosis were selected to generate a heatmap. The results showed that PB can regulate 15 genes associated with ferroptosis by regulating glutathione metastasis or iron ion transport, including TFR1 (TFRC), SLC7A5, GPx4, and SLC3A2 (Figure 6E-F).

### Proteomic analysis further confirmed that PB can regulate iron ion metabolism

**Figure 7:**
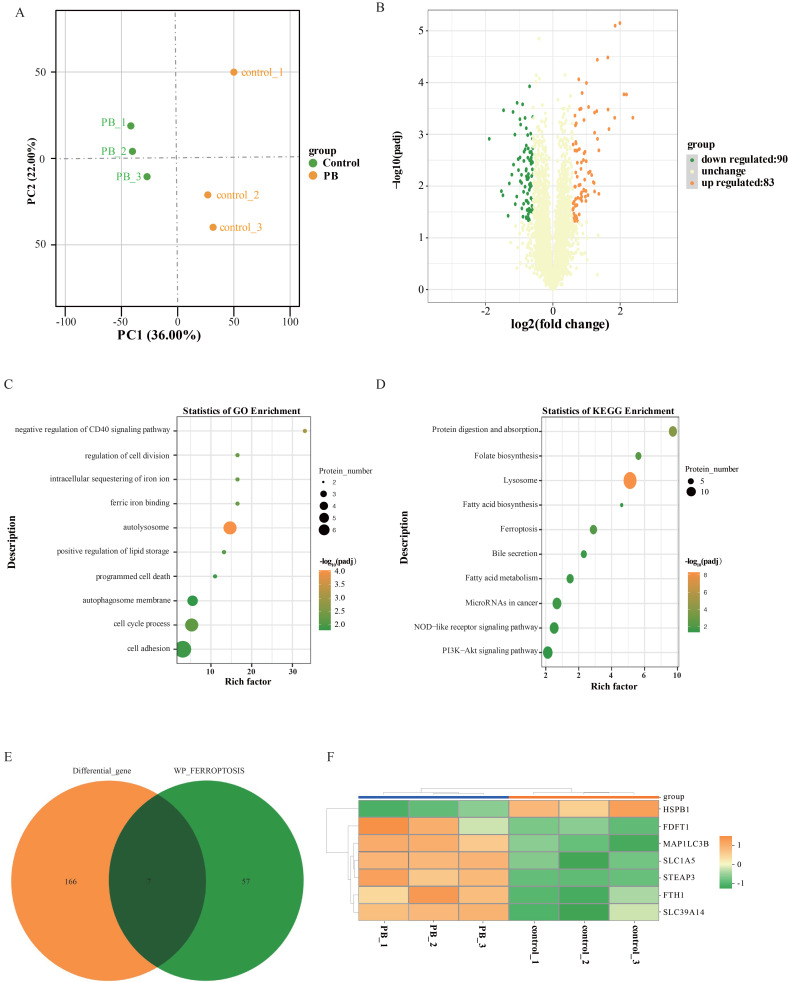
** Proteomic sequencing analysis after PB treatment in MKN-1 GC cells. A:** PCA of transcriptomes with or without PB treatment represented in a two-dimensional space. **B:** Volcano plot showed the differentially expressed proteins between PB-treated and untreated MKN-1 GC cells. **C-D:** GO analysis and KEGG analysis for the significantly differentially proteins after PB treatment in MKN-1 GC cells. **E-F:** Venn diagram and heat map to show the differentially expressed genes associated with ferroptosis.

Furthermore, quantitative proteome analysis was performed on MKN-1 GC cells after PB treatment using DMSO treatment. A total of 5718 proteins were identified and annotated in all samples. PCA showed that PC1 and PC2 in the PB group were transformed from those in the control group (Figure 7A). After preliminary screening, a total of 173 proteins were identified as differentially expressed proteins (Figure 7B). Compared with the control group, 83 proteins were upregulated and 90 proteins were downregulated in the PB group. To understand the effect of differential proteins on GC cells, GO and KEGG analyses were performed (Figure 7C-D). GO analysis showed that the differentially expressed proteins after PB treatment were mainly involved in autolysosomes, ferric iron binding, sequestration of iron ions, cell division, the cell cycle and cell adhesion, while KEGG analysis showed that PB could regulate lysosomes, ferroptosis, fatty acid metabolism, NOD-like receptor pathways and PI3K-Akt signalling. To fully understand the role of PB in regulating ferroptosis, the differentially expressed proteins associated with ferroptosis were selected to generate a heatmap. The results showed that PB can regulate 7 proteins associated with ferroptosis by regulating iron ion transport, including HSPB1, MAP1LC3B, and FTH1 (Figure 7E-F).

### Polyphyllin B can also induce ferroptosis via iron transport in GC cells *in vitro*

**Figure 8:**
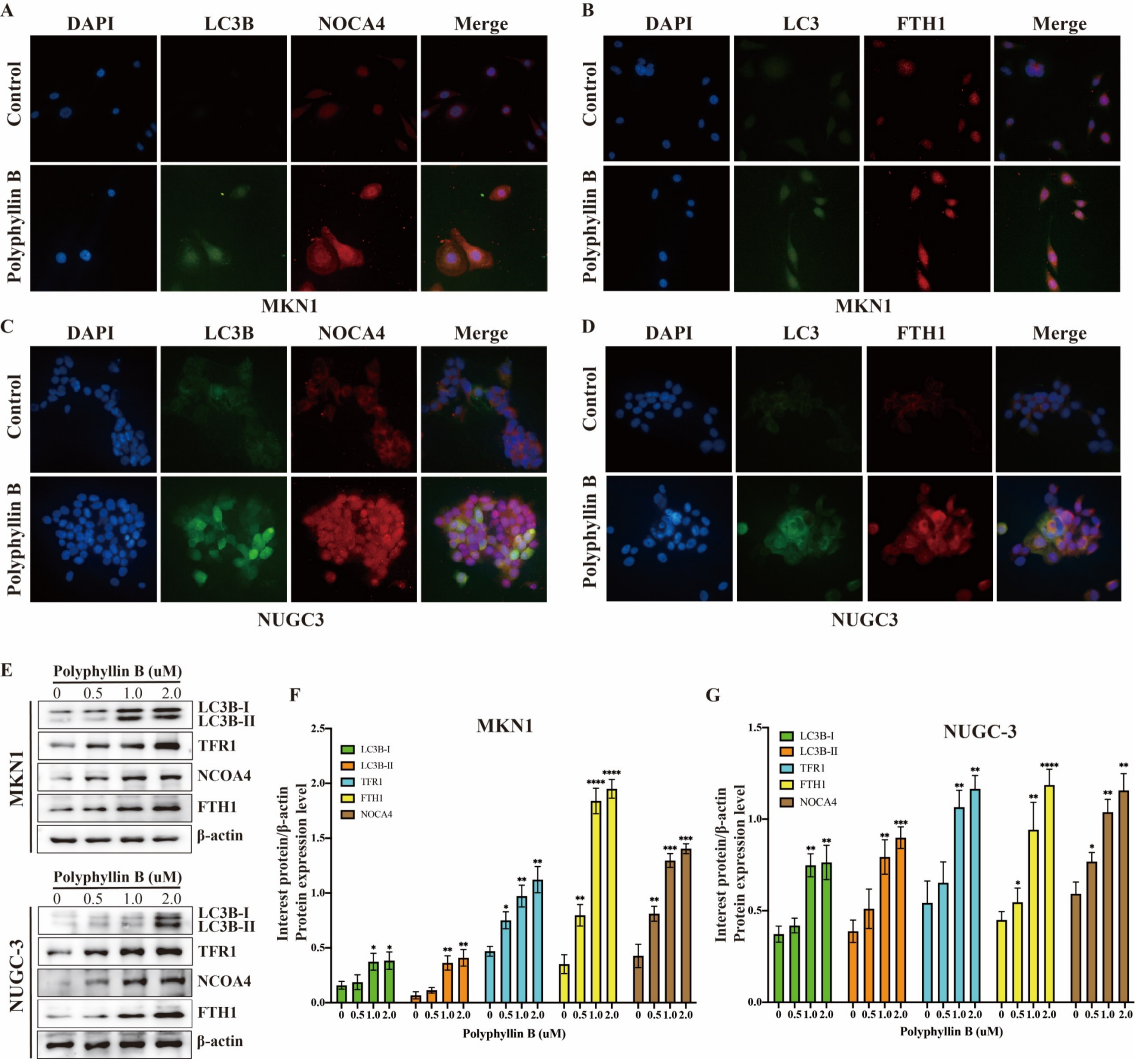
** PB regulates the iron metabolism in GC cells. A-D:** Immunofluorescence were used for examined the expression of LC3B, TFRC, NOCA4 and FTH1 after PB treatment in MKN-1 and NUGC-3 GC cells. **E-F:** Western blot were used for examined the expression of LC3B, TFRC, NOCA4 and FTH1 after PB treatment in MKN-1 and NUGC-3 GC cells, followed by quantitative analysis. * *P* < 0.05, ** *P* < 0.01, *** P < 0.001, **** P < 0.0001.

To examine whether PB can regulate iron transport in GC cells, the expression of TFRC, LC3B, NOCA4, and FTH1 was detected via immunofluorescence and western blotting (Figure 8). The results showed that the expression of TFRC, LC3B, NOCA4, and FTH1 was increased after PB treatment. These results suggest that PB may increase the level of Fe^2+^ by transporting Fe^3+^ into the cell by TFR1 and promoting NCOA4-dependent iron autophagy.

### Polyphyllin B suppresses tumor growth in an orthotopic mouse model of GC

**Figure 9:**
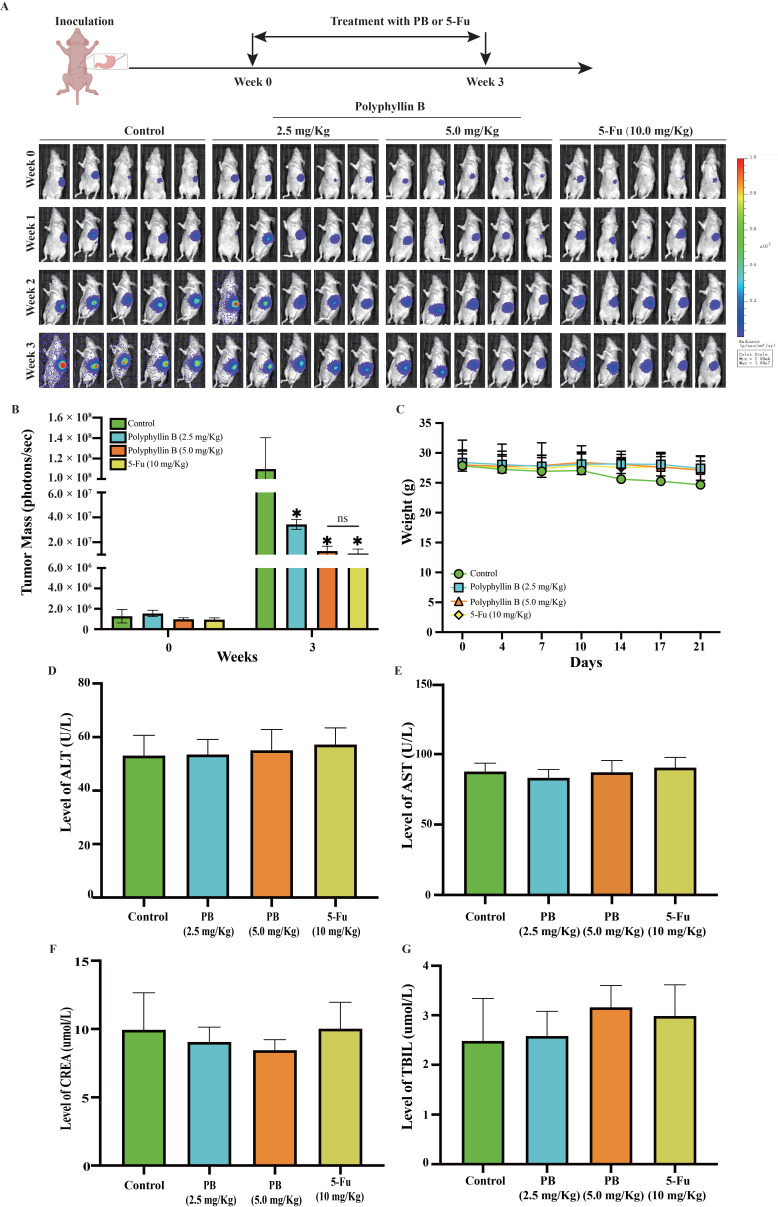
** PB inhibits the growth of MKN-1 orthotopic tumors without causing significant host toxicity.** Mice bearing MKN-1 orthotopic tumors were treated with PB by i.p. injection at the dose of 2.5 or 5.0 mg/kg/d or 5-Fu at the dose of 10 mg/kg/d, 7 days/week for 3 weeks. **A:** The luciferase signals in the mice were detected and images were obtained using an IVIS imaging system. **B:** At the end of the experiments, the average tumor mass (determined by the detected photons/sec) of the PB-treated mice was compared with vehicle-treated mice. **C:** The mice were monitored for changes in body weight as a surrogate marker for toxicity. **D-G:** The level of ALT, AST, TBIL, CREA in peripheral blood were detected after PB or 5-Fu treatment as markers for hepatic and renal function.

We assessed the efficacy of PB in an orthotopic mouse model of GC. Nude mice bearing orthotopic MKN-1 tumours were treated with PB by intraperitoneal injection at doses of 2.5 or 5.0 mg/kg/day or 5-Fu by intraperitoneal injection at doses of 10.0 mg/kg/day, 7 days/week for 3 weeks. As shown in Figures 9A-B, PB treatment at doses of 2.5 and 5.0 mg/kg/day significantly inhibited the growth of MKN-1 orthotopic tumors by 68.68% and 88.25%, while 5-Fu at 10 mg/kg/day significantly inhibited the growth of MKN-1 orthotopic tumors by 89.96%. And there was no significant difference between PB treatment at 5.0 mg/kg/day and 5-Fu treatment at 10 mg/kg/day. PB treatment at a dose of 2.5 or 5.0 mg/kg/day did not significantly affect the average body weight of the mice in comparison to that of vehicle-treated mice, and there was no obvious abnormality in the levels of ALT, AST, TBIL, and CREA in peripheral blood. This result suggested that PB at this effective dose did not cause significant host toxicity during the treatment period (Figure 9C-G).

**Figure 10:**
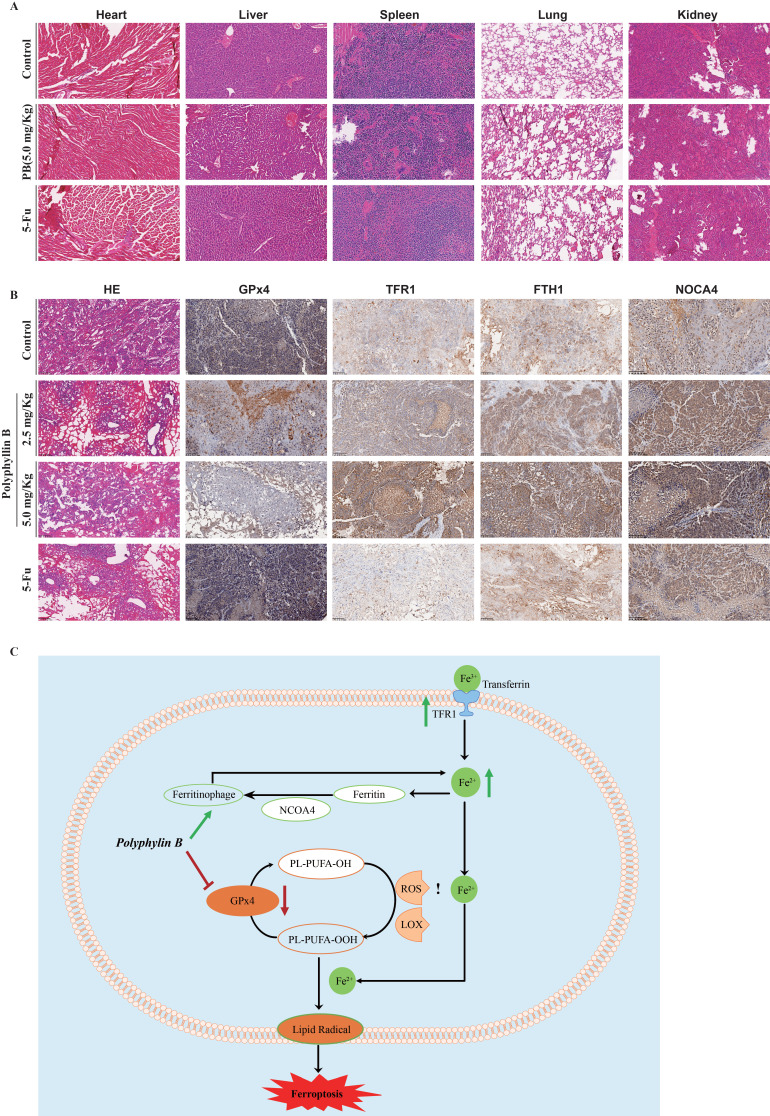
** PB regulates GPx4 expression and the iron metabolism *in vivo*. A:** Representative H&E staining images of various organs after PB or 5-Fu treatment. **B:** Representative immunohistochemical images of GPx4, TFR1, NOCA4 and FTH1 expression after PB or 5-Fu treatment. **C:** Scheme showing a proposed molecular mechanism for PB inducing ferroptosis.

Moreover, the expression of GPx4, TFR1, FTH1, and NOCA4 was detected via immunohistochemistry. The results showed that PB treatment inhibited GPx4 expression, while the expression of TFR1, NOCA4, and FTH1 was increased after PB treatment (Figure 10A-B). These results suggested that both GPx4 expression and iron ion metabolism play important roles in PB-induced ferroptosis.

## Discussion

GC is a common malignant tumour with poor prognosis, and new therapeutic targets are urgently needed to develop effective anti-GC drugs. Ferroptosis plays an important role in maintaining homeostasis, cell differentiation, growth and proliferation. It has different effects in different stages of tumorigenesis and development, co-regulated by various mechanisms such as genetics and transcription 18. Therefore, targeting pathways that regulate ferroptosis in tumour cells has become a new option for antitumour therapy.

GPx4 plays an important role in regulating ferroptosis and is generally considered to be a negative regulator. Guerriero et al. 19 showed that GPx4 expression in hepatocellular carcinoma tissues was obviously higher than that in paired adjacent tissues, and it was correlated with histological grade. Hangauer et al. 20 confirmed that the expression level of GPx4 was significantly increased in cancer cell lines from drug-resistant patients with lung cancer, ovarian cancer and breast cancer. However, the drug resistance of cancer cells was significantly reduced while downregulating the expression of GPx4, suggesting that GPx4 may reduce the sensitivity of cancer cells to radiotherapy and chemotherapy by regulating the intracellular oxidation level of tumour cells. TIMER database analysis showed that the mRNA expression levels of GPx4 were different between tumour and adjacent normal tissues in a pan-cancer dataset, and GPx4 was overexpressed in stomach adenocarcinoma compared with adjacent normal tissues (P<0.01). Patients with high GPx4 expression had a poorer prognosis than those with low GPx4 expression. Our clinical sample also confirmed that the expression of GPx4 is gradually increased in normal tissues, intestinal metaplasia, dysplasia, early GC, and advanced GC. However, additional clinical samples from multicentre centres are needed for verification, and some important clinicopathological features should also be considered. Recently, targeting GPx4 remains an active area of drug development in cancer therapy. RSL3, the first inhibitor found to directly inhibit GPx4, contains an electrophilic chloroacetamide that binds irreversibly to its active site, leading to GPx4 inactivation. Similar to RSL3, ML162 and DPI also lead to GPx4 inactivation by covalently binding to GPx4. FIN56 can degrade GPx4 proteins 21, while FINO2 can indirectly inactivate GPx4 and oxidize iron ions 22. However, most of the existing GPx4 inhibitors have poor pharmacokinetic properties, which limits their clinical application.

Traditional Chinese medicine (TCM) originated from long-term clinical practice under the guidance of the corresponding theoretical system. It has a definite effect in disease intervention and is an important source of modern innovative drug research and development. Here, we identified a novel GPx4 inhibitor, PB, a dioscin isolated from *Paris formosana* Hayata plants, which has been used as the main component of traditional anticancer medicine for treating lung cancer, gastric cancer and other tumours 23. A previous study found that PB had antitumour activity, which may be related to the regulation of the immune response 24, 25. This study demonstrated that PB can effectively inhibit the proliferation of GC cells, induce ferroptosis and apoptosis, and prevent migration, invasion and the cell cycle *in vitro*. In addition, PB inhibited pancreatic tumour growth *in vivo* without causing any significant host toxicity. And there was no significant difference in tumor-suppressive effect between PB treatment at high concentration and 5-Fu treatment. Mechanistically, PB may directly bind to GPx4 and reduce the expression of GPx4, while it can also regulate iron ion metabolism by promoting iron ion transport and ferritinophagy, which ultimately leads to ferroptosis. However, we found that the mRNA expression of TFR and GPx4 was opposite to the protein expression, which may be a negative feedback regulation process. In addition, the anti-GC function of PB may also be associated with its regulation of the MAPK signalling pathway, PI3K-Akt signalling pathway and TNF signalling pathway. However, further exploration is needed to determine whether PB also inhibits GC through these pathways, and PB may have secondary molecular targets that should also be investigated in the future.

## Conclusion

In this study, we identified a novel GPx4 inhibitor, PB, which shows good antitumor efficacy without causing significant host toxicity via inducing ferroptosis in both gastric cancer cells and mouse models. In conclusion, our results confirmed that GPx4 may be a potential target for GC treatment, we also provide a novel and promising drug for the treatment of GC.

## Supplementary Material

Supplementary raw data.

## Abbreviations

GC: Gastric cancer
PB: Polyphyllin B
IARC: International Agency for Research on Cancer
GPx4: Glutathione peroxidase 4
GSH: Glutathione
