# Supplementary material for: Polyphyllin B Suppresses Gastric Tumor Growth by Modulating Iron Metabolism and Inducing Ferroptosis
Source: Int J Biol Sci. 2023 Jan 31;19(4):1063–79. doi: 10.7150/ijbs.80324 (PMC10008684; doi:10.7150/ijbs.80324)

Polyphyllin B suppresses gastric tumor growth by modulating iron metabolism and inducing ferroptosis

Uncut orginal image

Figure-2E

MKN-1

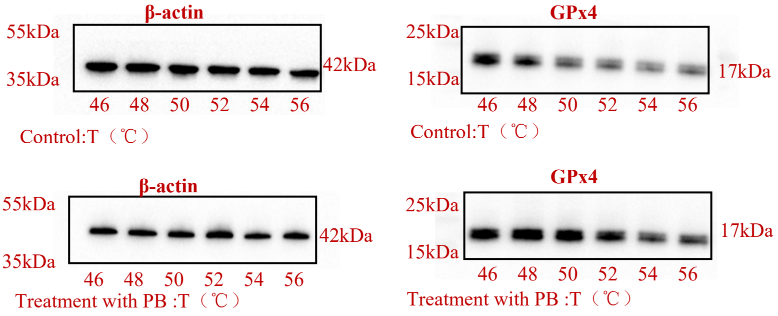

NUGC-3

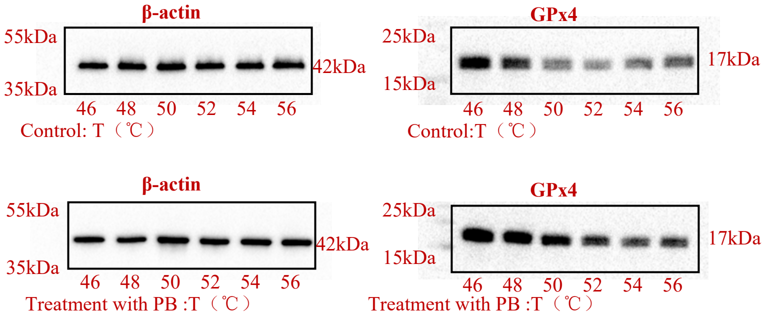

Figure-2F

MKN-1

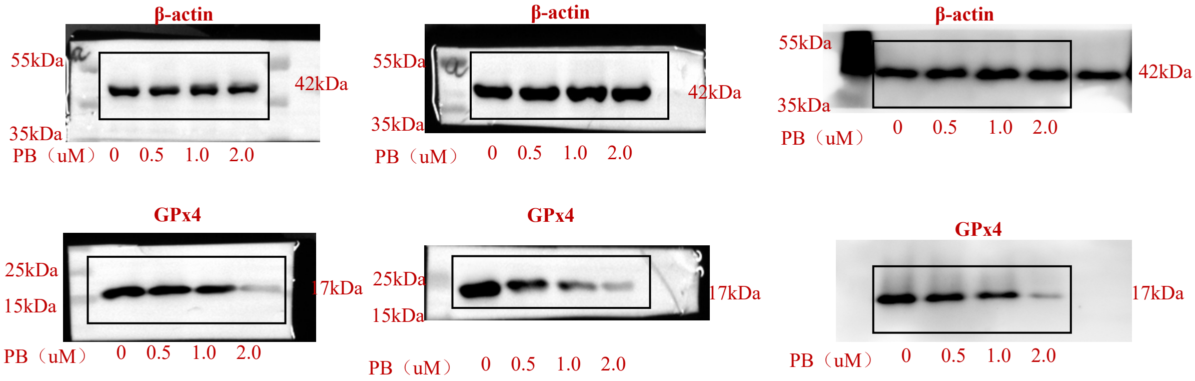

NUGC-3

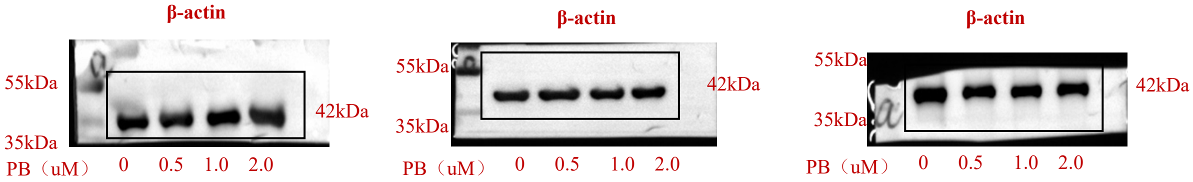

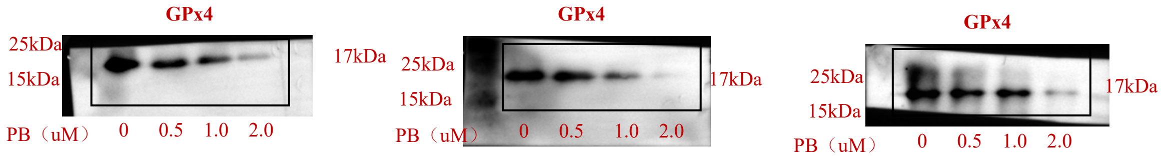

Figure-4G

MKN-1

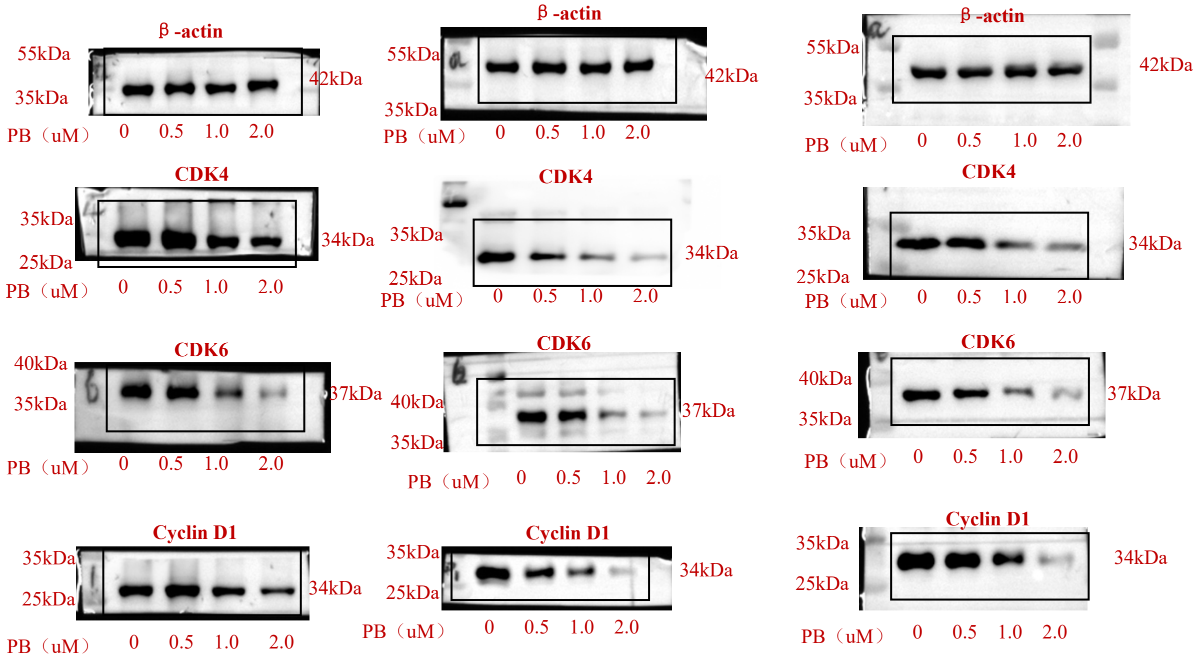

NUGC-3

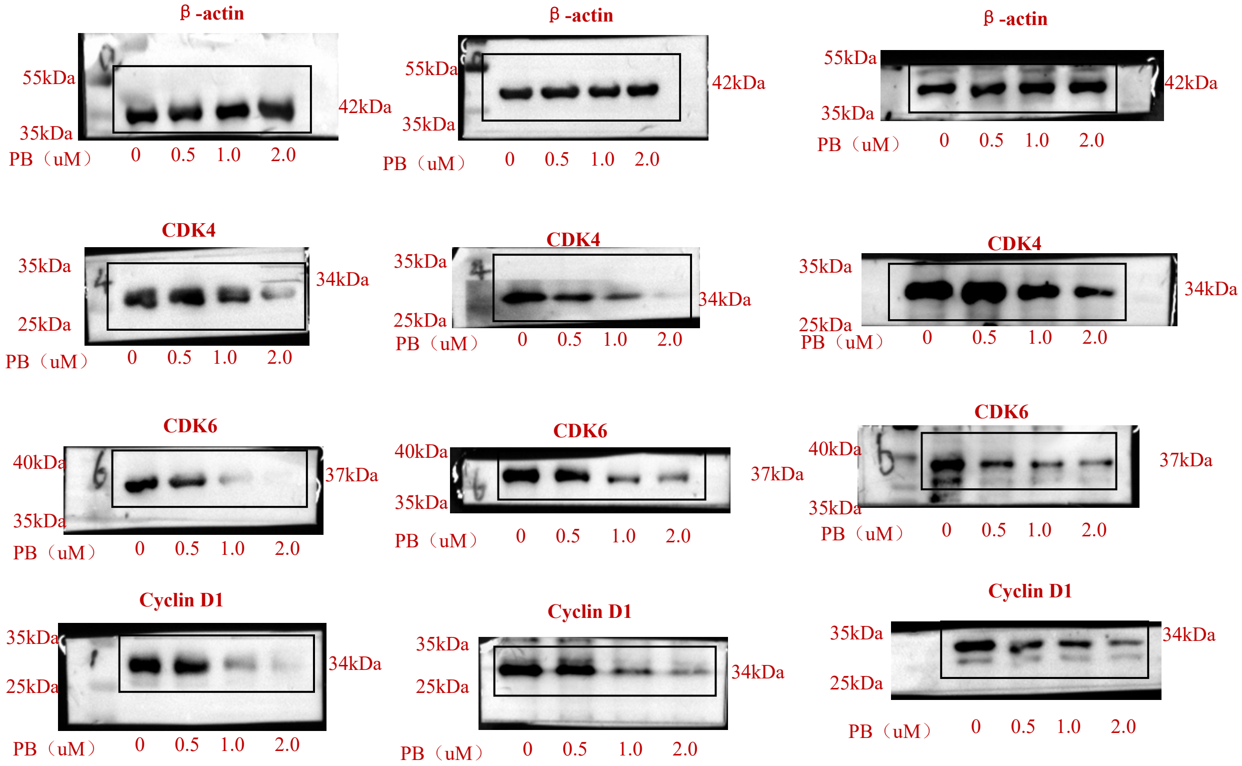

Figure 8 E

MKN-1

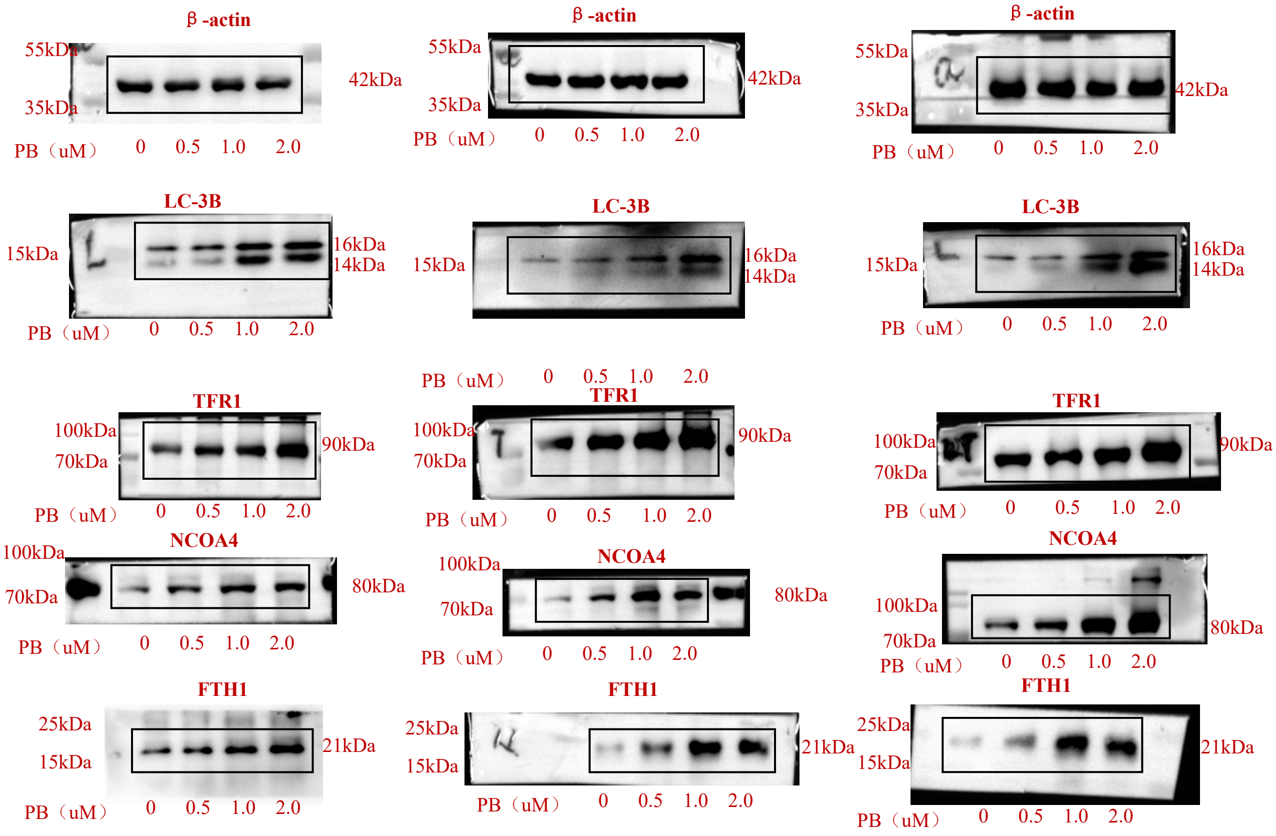

NUGC-3

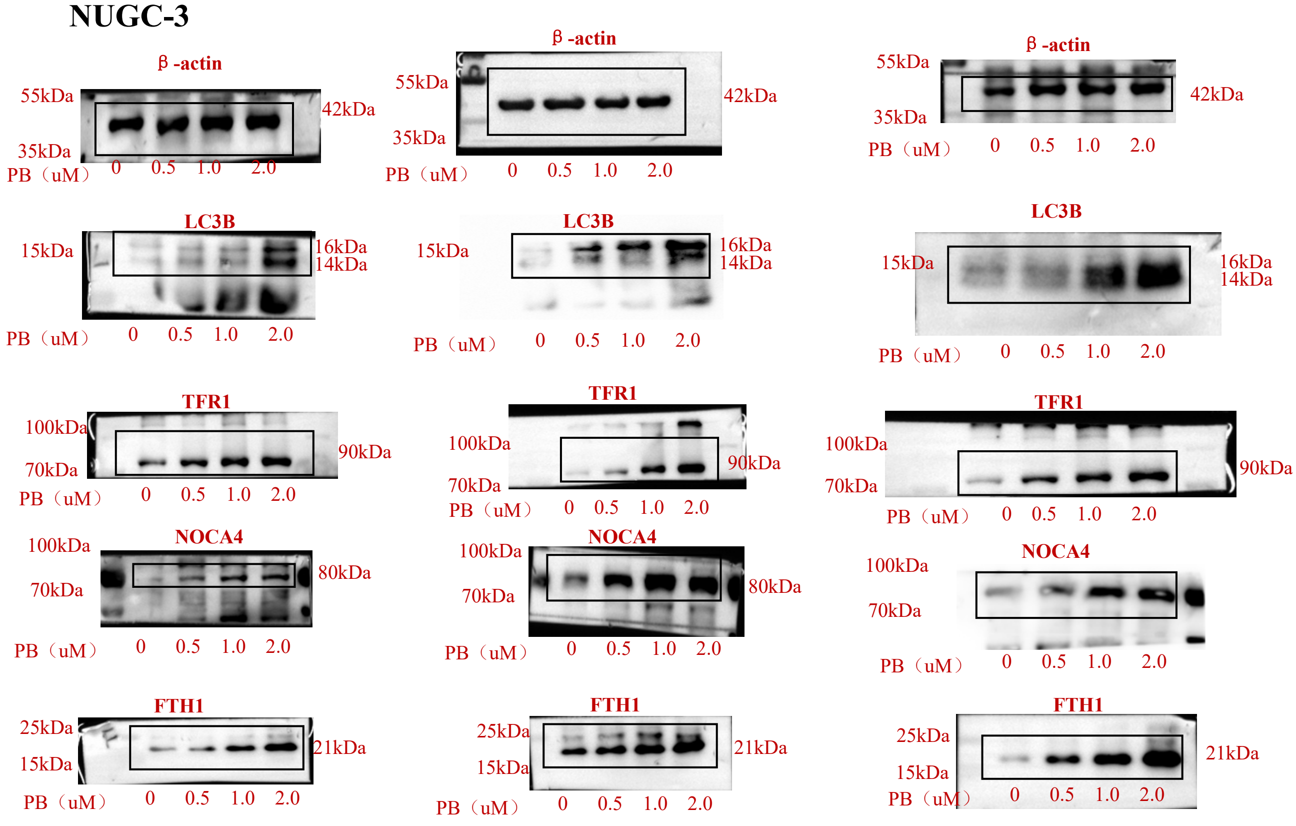

Supplement: Supplementary file 1 — Supplementary raw data. [file ijbsv19p1063s1.pdf]
